# Dynamic Stability Analysis of Progressive Supranuclear Palsy Affected Gait Using Lyapunov Floquet Theory

**DOI:** 10.1109/TNSRE.2025.3614555

**Published:** 2025

**Authors:** Sandesh G. Bhat, Farwa Ali, Cecilia A. Hogen, Asghar Rezaei, Keith A. Josephs, Jennifer L. Whitwell, Kenton R. Kaufman

**Affiliations:** Motion Analysis Laboratory, Division of Orthopedic Surgery, Mayo Clinic, Rochester, MN 55905 USA; Department of Neurology, Mayo Clinic, Rochester, MN 55905 USA; Motion Analysis Laboratory, Division of Orthopedic Surgery, Mayo Clinic, Rochester, MN 55905 USA; Department of Physiology and Biomedical Engineering, Mayo Clinic, Rochester, MN 55905 USA; Department of Neurology, Mayo Clinic, Rochester, MN 55905 USA; Division of Neuroradiology, Department of Radiology, Mayo Clinic, Rochester, MN 55905 USA; Motion Analysis Laboratory, Division of Orthopedic Surgery, Mayo Clinic, Rochester, MN 55905 USA

**Keywords:** Progressive supranuclear palsy, parkinsonism, dynamic balance, gait disorders, nonlinear dynamics, assessment in rehabilitation

## Abstract

Progressive supranuclear palsy (PSP) is a neurodegenerative disease with severe gait and balance deficits. There are no effective ways to assess dynamic balance during walking in PSP. The Lyapunov Floquet (LF) theory has been utilized to study dynamic balance in healthy and pathologic gait but has not been applied to PSP affected gait. In the current study, the medio-lateral motion of the center of mass during gait for 40 patients with PSP (PSP group) and 33 healthy older adults (Control group) were studied. Metrics from LF theory, such as the maximal Floquet multiplier (FM), maximal long-term Lyapunov Exponent (LE_L_), and maximal short-term Lyapunov Exponent (LE_S_) were used to study walking stability. Although all the gait dynamics for all the participants were stable and non-chaotic, the PSP group was observed to be closer to an unstable system and more susceptible to perturbations (|FM| closer to 1 and LE_L_ closer to 0) than the Control group (p < 0.001). The control group’s stability deteriorated, and the gait system became more susceptible to perturbations with age. Such a trend was not observed in the PSP group. The risk of falls increased with increase in cadence in the PSP group (p < 0.001). These findings demonstrate the potential of LF theory measures to evaluate dynamic stability in patients with PSP and the need for future research using quantitative measures.

## I. Introduction

Progressive Supranuclear Palsy (PSP) is a rapidly progressing, fatal neurodegenerative disease characterized by early onset of severe gait and balance abnormalities [2], [3]. The approximate age of disease onset for PSP is 40 years and older [4], [5]. PSP is often misdiagnosed as Parkinson’s Disease (PD) due to the similar symptoms and disease presentations especially early in the disease course [6], [7]. One of the cardinal features that distinguish PSP from other parkinsonisms is early onset of postural instability leading to falls. Quantified gait analysis has been used to detect gait and balance abnormalities in PSP that may not be evident on clinical neurologic exam [8], [9]. Spatial temporal parameters show slow velocity, short stride length, reduced cadence and increased support times [10]. Balance abnormalities have focused on sway, which is a measure of static balance that is worse in PSP than PD and controls [11], [12]. Double support time during stance phase has been used by some as a surrogate of dynamic stability but does not offer a true evaluation of stability during walking [8]. Dynamic instability is a prominent early clinical feature in PSP which contributes to falls, disability and loss of independence [13]. Hence, an objective measure of dynamic balance of PSP affected gait is required and could provide early and accurate diagnosis. In our research, we investigate Lyapunov Floquet (LF) theory metrics as a measure of dynamic stability in PSP.

The dynamics of human walking can be approximated as a periodic time varying system. Dynamical system analysis techniques, like the LF theory, allow for the analysis of dynamic stability and the chaotic nature of such an approximated system. Metrics, such as the Lyapunov exponents (LE) and the Floquet multipliers (FM), derived from the LF theory have been applied to quantify dynamic stability during walking [14], [15], in neurological gait disorders such as multiple sclerosis [16] and cerebellar ataxia [17], and have been shown to identify fall-prone individuals [18], [19]. A dynamical system is considered stable if the absolute value of FM ≤ 1 [20]. The LE is used to analyze the chaotic behaviour of dynamical systems and the rate at which the system approaches/diverges from its attractor/repeller respectively. In a dynamical system representing gait, an attractor represents a state of stability (e.g., stable walking or stand still) toward which the gait dynamics naturally converge over time. Conversely, a repeller corresponds to a state of unstable gait pattern (e.g., falls) from which the system diverges, highlighting instability or unsteady behavior in walking. A system with LE ≤ 0 is considered stable. In a simulation to examine the stability of human walking, Bruijn et al. showed that LE when calculated over 0.5 gait cycle (LE_S_) may be a valid predictor of global gait stability [21]. Su and Dingwell observed that in a simulated walking model with perturbations, an increase in LE_S_ captured the increased risk of falling [22]. Similarly in human gait studies, Kurz et al. studied gait stability (amount of divergence in the attractor’s dynamics during walking) using LE, and observed that the LE was greater in patients with idiopathic PD compared to younger healthy individuals [23]. Another human gait study performed to measure dynamic stability showed that LE when calculated over 10 gait cycles (LE_L_) was more sensitive to gait instability in the medio-lateral (M/L) direction [24].

In this study, we investigated an application of the LF theory to PSP affected gait to identify a measure of gait instability. To our knowledge, such an application has not been successfully defined previously. The primary hypothesis tested was that the metrics derived from the LF theory that describe the stability and chaotic behavior of the system (LE_L_, LE_S_, and FM) would be different for PSP affected gait compared to healthy individuals within a similar age range.

## II. Methods

### A. Participants

The patients with PSP were recruited from the Neurodegenerative Research Group (NRG) in the Department of Neurology, Mayo Clinic, Rochester, MN (NIH NINDS R01 NS089757 AND K23 NS124688). Study participants fulfilled the 2017 Movement Disorder Society clinical criteria for PSP [5] and were able to walk with or without assistance. Exclusion criteria were immobility due to advanced disease stage or an alternative cause of gait impairment such as Parkinson’s disease, severe degenerative arthritis, or amputation.

For the control group, healthy participants (above 50 years of age, community dwelling, without any diseases/conditions that affected their gait) were recruited from the Rochester, MN community using solicitation methods such as flyers, reaching out to individuals from previous studies, and the Rochester-area Older Adult Registry (ROAR) [25]. ROAR is a Mayo Clinic community-based, longitudinal primary care population of adults ≥ 65 years of age that collects information to address scientific questions on determinants of healthy aging. The healthy participants in the current study did not undergo a lower extremity surgery and had normal neuromuscular exam.

Informed consent was obtained prior to data collection under the guidelines set by Mayo Clinic’s institutional review board (No. 20-013160, 23-004889, and 15-004618).

### B. Data Collection

All data collection was conducted in the Motion Analysis Laboratory, Mayo Clinic, Rochester, MN. Demographic data, such as age, height, and body mass were collected initially. A neurologist measured disease severity within the PSP group using the progressive supranuclear palsy rating scale (PSPRS), and PSPRS gait midline (PSPRS-GM). All participants were instructed to walk barefoot at a comfortable pace on a 10m level ground walkway for a minimum of 5 consecutive trials. Participants in the PSP group were secured in a ceiling-mounted, fall-safety harness system (harness vest shoulder straps adjusted such that participants could freely achieve 15-degrees of trunk flexion from standing) for all trials. Since patients did not use assistive devices or therapist assistance during testing, the safety-harness did not influence our findings. Three-dimensional trajectory data for retroreflective markers placed on the bilateral heels (RHEE, LHEE), and posterior and anterior iliac crests (RPSI, LPSI, RASI, and LASI) were collected at 120Hz with a 14-camera motion capture system (Raptor 12HS, Motion Analysis Corporation, Rohnert Park, CA) for all trials. The first and last two strides for each trial were omitted to account for gait initiation and termination, resulting in an average of 19 total strides being analyzed over 5 trials. The timeseries data were further processed to eliminate frames with incomplete data due to poor marker visibility and were exported for analysis. Collection of clinical data and the gait study were performed on the same or adjacent days.

### C. Data Processing

**Fig. 1.**
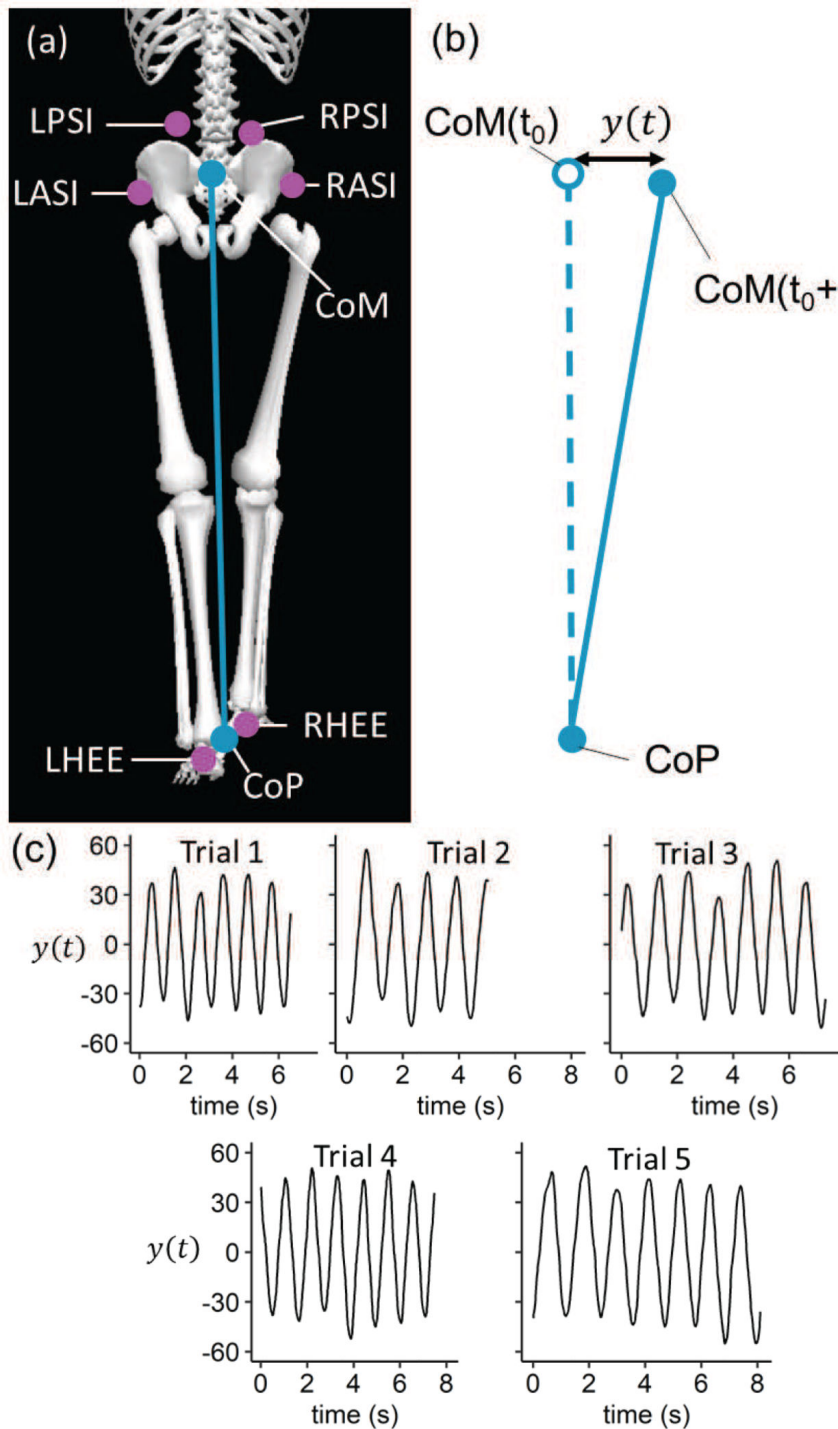
(a) Frontal plane representation of the subject during gait, (b) Representation of the calculation of y(t) using the position of the CoM relative to the CoP, (c) Mean normalized medio-lateral (M/L) trajectory of the CoM for 5 trails. The movement of the CoM was similar for each trial. LPSI: Left superior posterior iliac spine; RPSI: Right superior posterior iliac spine; LASI: Left superior anterior iliac spines; RASI: Right superior anterior iliac spines; CoM: surrogate center of mass; CoP: surrogate center of pressure. [1].

The average of the RPSI, LPSI, RASI, and LASI markers in the M/L direction was considered a surrogate for the M/L movement of the center of mass (CoM) and the average of the RHEE and LHEE markers in the M/L was considered a surrogate for the center of pressure (CoP) [26] (Fig 1 (a)).

**Fig. 2.**
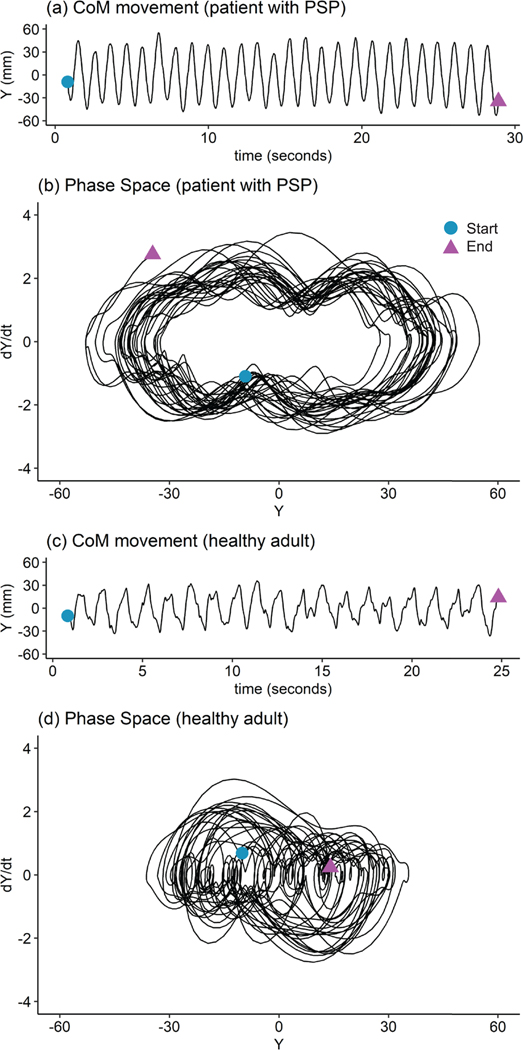
Representative CoM data for a participant with PSP and a healthy participant. (a) and (c) Stitched and filtered CoM data; (b) and (d) Phase space representation of the system with the CoM data on the horizontal axis, and the derivative of the CoM data on the vertical axis. The start and end points are marked on both the graphs for reference.

The trajectory of the CoM relative to the CoP in the M/L direction $\left(y(t)=CoM_{ML}-CoP_{ML}\right)$ was calculated and the averaged value of the trajectory was subtracted (mean normalization). All 5 trials were stitched together and filtered for noise (lowpass Butterworth filter with a cutoff of 10 Hz [27]) to form a longer dataset (Y(t)) (representative example in Fig 2 (a), (c)) [1]. The application of LF theory to a stitched data set was validated by Sloot et al. [28] and a previous study [1]. A phase space representation for the system was defined as $m(t)=[Y(t),\dot{Y(t)}]$ where $\dot{Y(t)}$ is the time derivative of Y(t) (graphical representation in Fig 2 (b), (d)). Using an optimized time-delayed embedding, system estimation was performed to obtain the system M(t)=[m(t),m(t+τ),m(t+2τ),…,m(t+nτ)] where τ was the time delay, and n was the embedding dimension [1]. The monodromy matrix for the system was calculated as $\text{Φ}(T)=M(T)M^{-1}(0)$. The eigen values of the monodromy matrix were the FM. The eigenvalues of R, where R=(log(Φ(T))/T), were the Floquet exponents. The real parts of the Floquet exponents were the LE_L_. Short term LE (LE_S_) was calculated using Rosenstein et al’s algorithm [29]. The mean values of the FM, the maximal values of the LE_L_ and the maximal values of the LE_S_ were used to compare the stability and the behavior of the system under perturbations [1]. An LE > 0 indicated poor perturbation tolerance and the system was termed chaotic, and a FM > 1 indicated an unstable system. All calculations were performed in MATLAB 2021b (MathWorks, Natick, MA).

### D. Statistics

The participant’s demographics data (age, height, body mass, and cadence) were analyzed using a Welch two sample t-test for any differences between the groups (Control vs. PSP). The normality assumption was tested using the Anderson-Darling test. An ANCOVA test was used to analyze the differences in the dependent variables (maximal LE_L_, maximal LE_S_, and mean FM) between the Control and PSP groups. The assumptions for ANCOVA were tested using the Anderson-Darling test (for normality of residuals) and the Levene’s test (for homogeneity of variances). Any demographics variables found to be different between the groups, along with sex, were used as the covariates in the ANCOVA test. Any covariates that had a significant effect on the dependent variables were used in a linear regression for post-hoc comparison. Spearman’s rank correlation test was used to study the correlation between the LF metrics (maximal LE_L_, maximal LE_S_, and mean FM) and the disease severity measures (PSPRS and PSPRS-GM). Statistical significance was set at p < 0.05. All analyses were performed using RStudio [30].

## III. Results

A total of 48 patients with PSP and 40 healthy individuals were recruited. Due to technical issues during data collection, only the data for 40 patients with a PSP (PSP group) and 33 healthy individuals (Control group) could be used for the current study. All participants in the study walked without the use of assistive devices or assistance from the study staff. The PSP and Control groups were similar in height and body mass. The participants in the PSP group were 7 years older (p <0.001) and took 5 less steps per minute (p < 0.001) than the participants in the Control group (Table I). Hence, sex, age and cadence were considered covariates for the ANCOVA tests. Additionally, none of the participants in the PSP groups fell during data collection.

### A. ANCOVA Tests

**Fig. 3.**
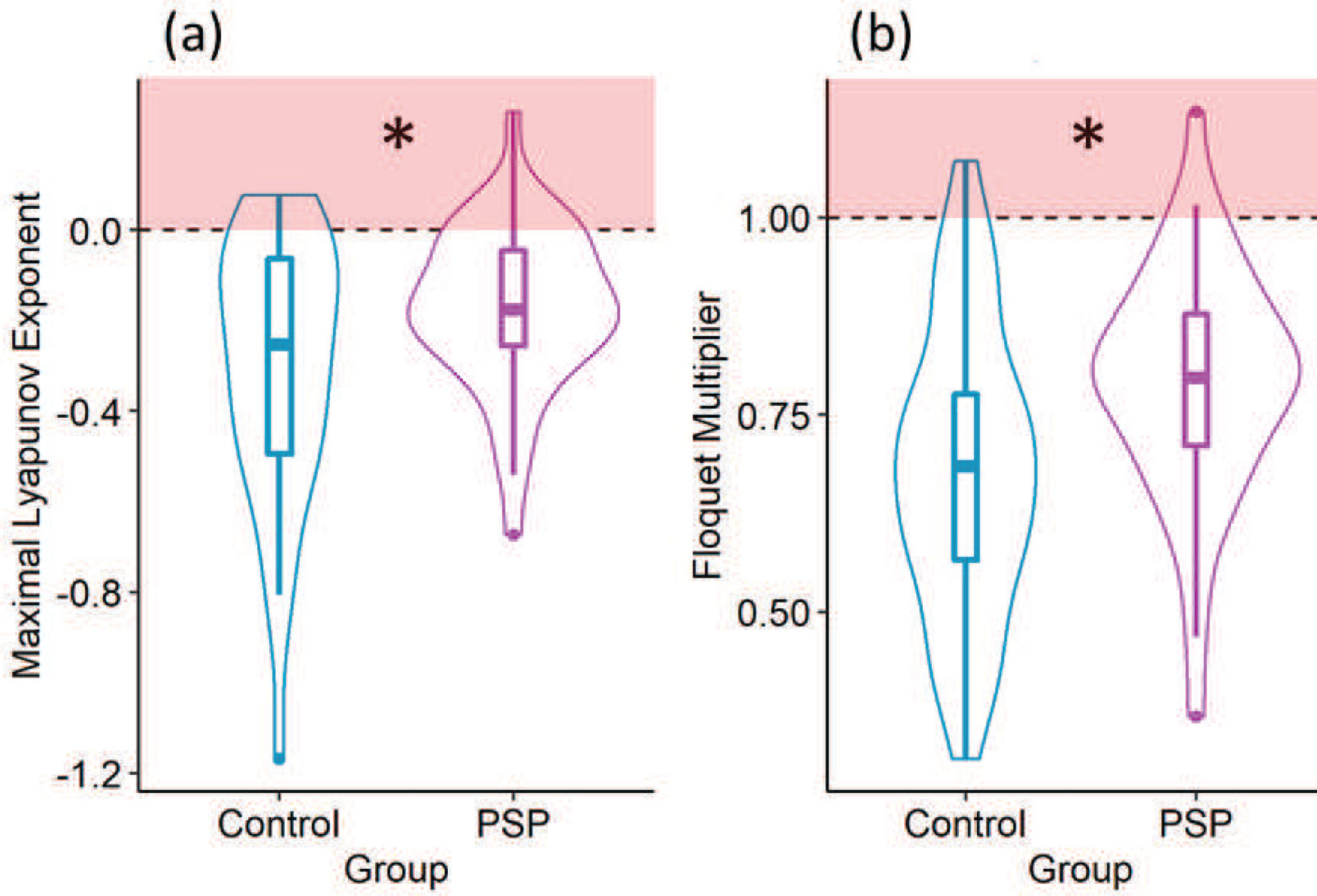
Violin plot and boxplots for the Control and PSP groups. (a) The maximal LE_L_ was greater for the PSP group than the Control group (p < 0.001). The red shaded area indicates a chaotic system. (b) The mean FM was greater for the PSP group than the Control group (p < 0.001). The red shaded area indicates an unstable system. Statistical significance is denoted by an asterisk (*).

**Fig. 4.**
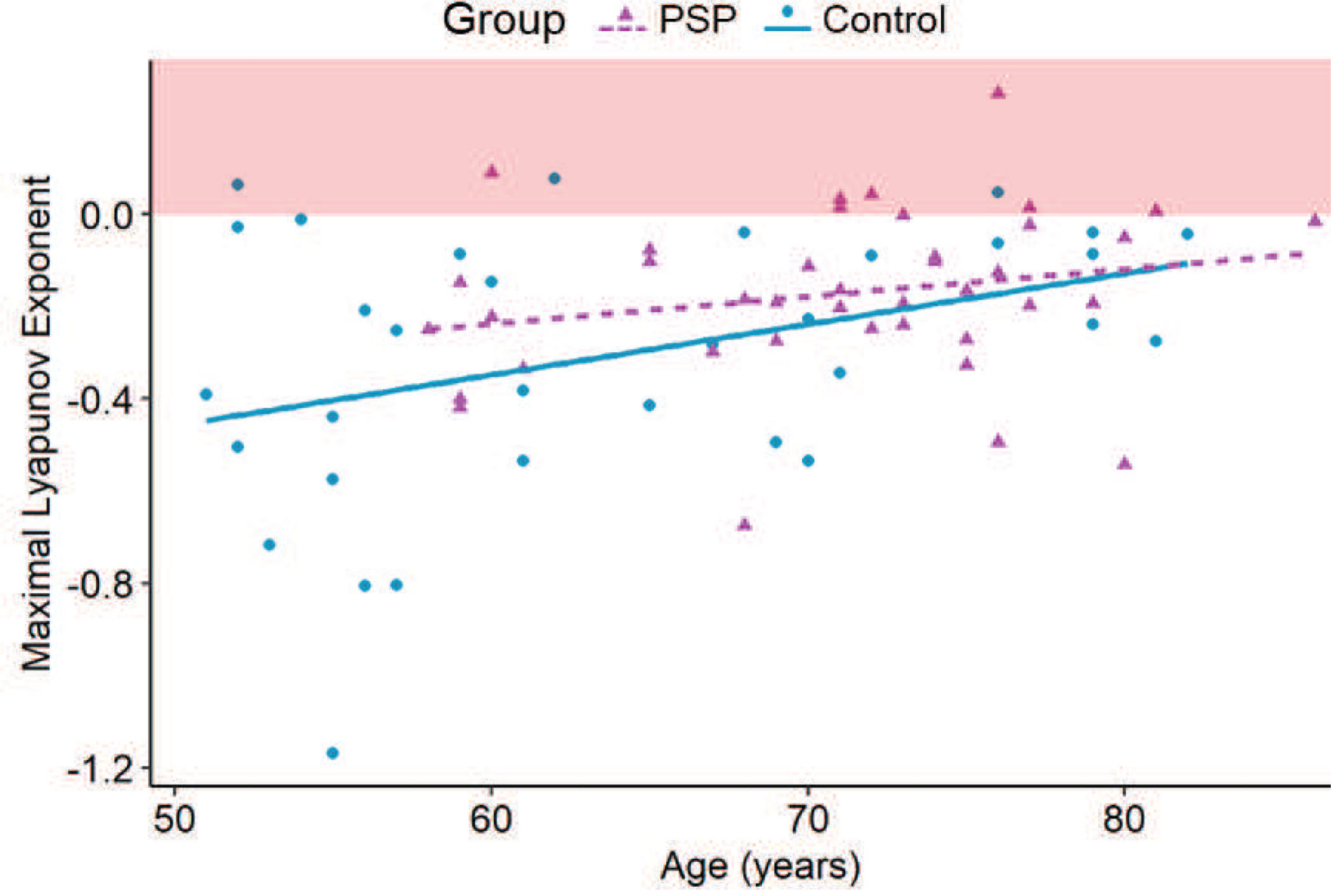
Post-hoc linear regression analysis performed on the Control and PSP groups. The maximal LE_L_ for the Control group increased with age (p = 0.03), but such a relationship was not observed in the PSP group (p > 0.05). The red shaded area theoretically indicates a chaotic system.

The behavior of PSP affected gait was closer to a chaotic system (maximal LE_L_ = −0.17 ± 0.18) compared to that of healthy gait (maximal LE_L_ = −0.3 ± 0.29) (p = 0.015) (Fig 3 (a)). The values of maximal LE_L_ indicated that PSP affected gait was more susceptible to perturbation compared to healthy gait. Cadence and sex did not significantly affect the value of maximal LE_L_ of either the PSP affected gait or healthy gait (p > 0.05). The value of maximal LE_L_ reduced with age (p = 0.005) (Fig 4). The maximal LE_L_ for all the participants were less than zero. Hence, the gait for the studied PSP participants and Controls was chaotic in nature, with the PSP gait being more chaotic.

**Fig. 5.**
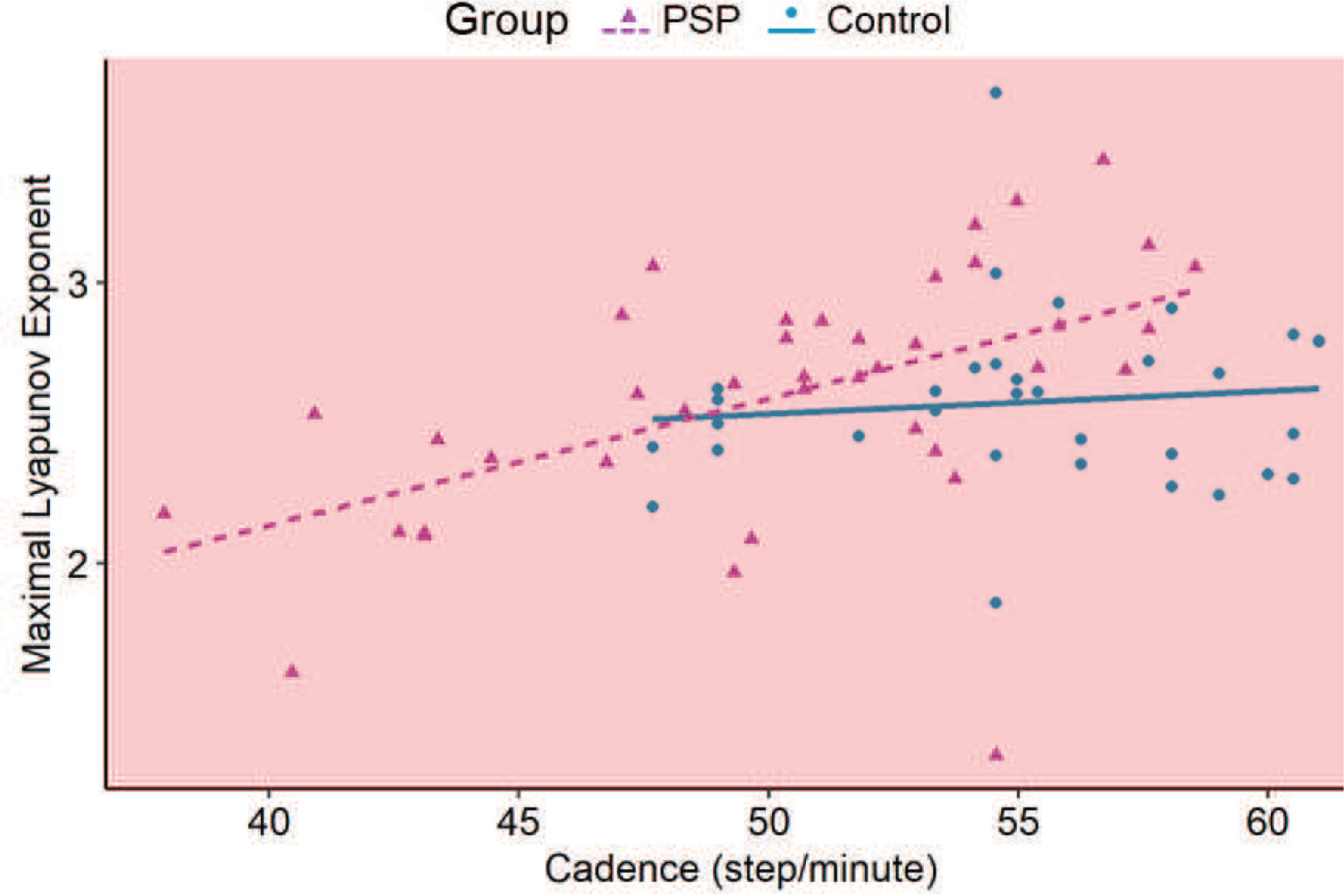
Post-hoc linear regression analysis performed on the Control and PSP groups. The maximal LE_S_ for the control group did not change with cadence (p = 0.55). The maximal LE_S_ for the PSP group increased with cadence (p < 0.001), with the risk of falling increasing with cadence. The red shaded area theoretically indicates a chaotic system.

Short term LE was similar for both groups (Control group: 2.58 ± 0.31; PSP group: 2.6 ± 0.44; p = 0.74). The participant’s age and sex did not affect this metric (p > 0.05) while the risk of falling, as measured by LE_S_, was significantly increased with increasing cadence (p < 0.001) (Fig 5). This indicated that LE_S_ was not able to reliably distinguish fall risk among PSP and control participants.

**Fig. 6.**
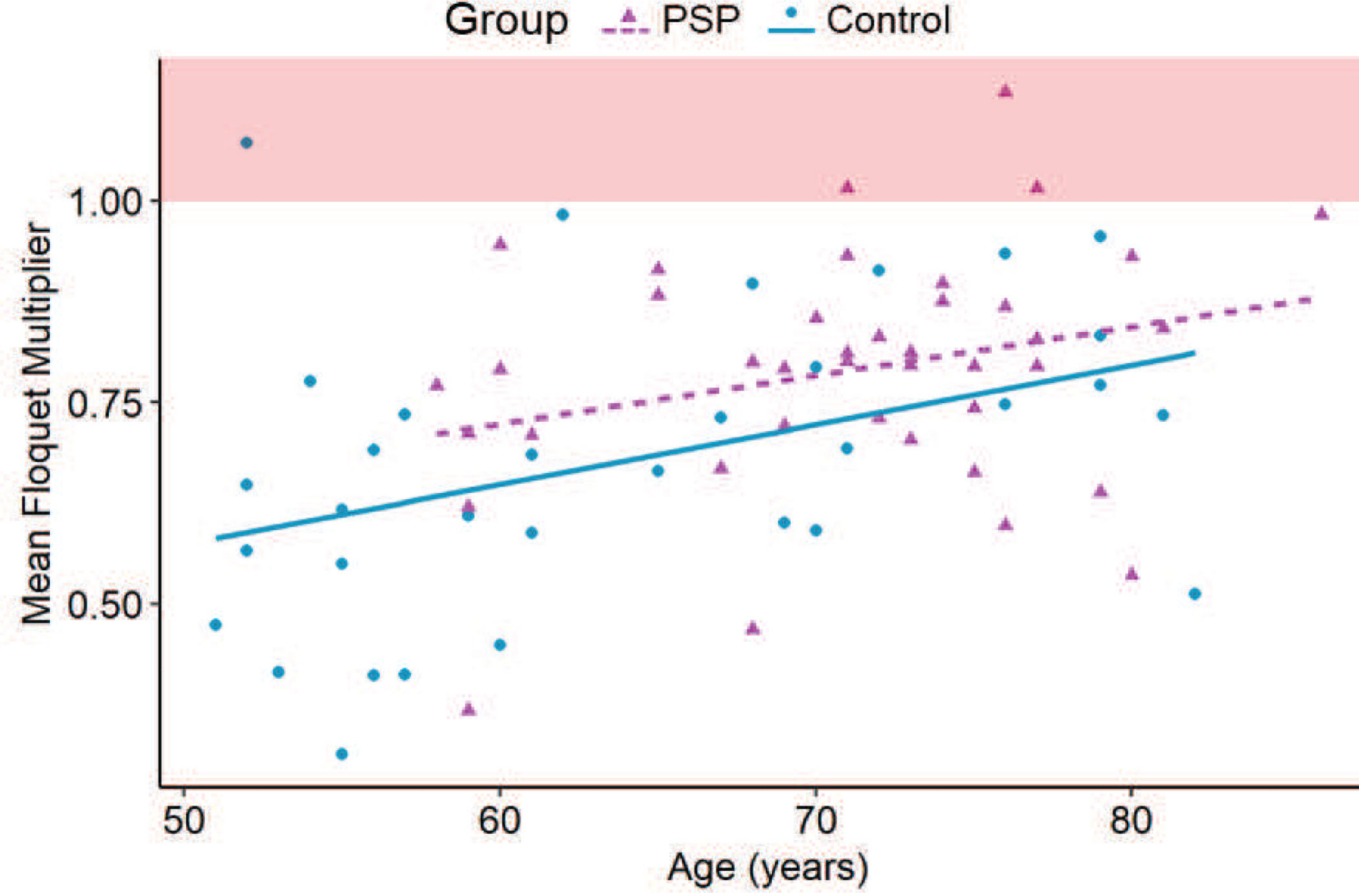
Post-hoc linear regression analysis performed on the Control and PSP groups. The mean FM for the Control group increased with age (p = 0.02), but such a relationship was not observed in the PSP group (p > 0.05). The red shaded area theoretically indicates an unstable system.

The FM for PSP affected gait (0.79 ± 0.15) was closer to the condition of instability compared to healthy gait (0.68 ± 0.18) (p < 0.001) (Fig 3(b)). Cadence and sex did not significantly affect the stability of either the PSP affected gait or healthy gait (p > 0.05), but age affected the stability significantly (p = 0.003) (Fig 6). Therefore, the FM indicated local instability in PSP affected gait.

### B. Regression Tests

Age significantly affected the maximal LE_L_. Linear regression analysis was performed post-hoc to study the relationship between age and maximal LE_L_ and stability. The gait in the control group became less tolerant to perturbation with age (slope = 0.1 per decade; R^2^ = 0.11; p = 0.03) (Fig 4). The effect of age on the perturbation tolerance of PSP affected gait was absent (slope = 0.06 per decade; R^2^ = 0.03; p > 0.05) (Fig 4). Therefore, PSP affected gait stability independent of age.

Cadence was a significant factor in the increase of risk of falling as measured by LE_S_. Post-hoc linear regression analysis showed that risk of falling did not change for the healthy participants (slope = 0.04 per 5 step/min; R^2^ = 0.01; p = 0.55) but increased significantly for the participants affected by PSP (slope = 0.23 per 5 step/min; R^2^ = 0.27; p < 0.001) (Fig 5). The participants affected by PSP had a greater range of cadence than the healthy participants, with the slowest participant with a PSP walking at only 38 steps/minutes. Hence, the participants in the PSP group had a greater risk of falling the faster they walked, suggesting dynamic instability.

A significant effect of age was also observed on the mean FM. The gait stability in the control group deteriorated with age (slope = 0.01 per decade; R^2^ = 0.13; p = 0.02) (Fig 6). Gait stability in the patients affected by PSP did not change with age (slope = 0.01 per decade; R^2^ = 0.05; p > 0.05) (Fig 6). Therefore, for healthy gait, the system’s stability reduced, and its behavior became more susceptible to perturbation with age, but such a trend was absent in the PSP affected gait.

### C. Correlation Tests

The LF metrics and disease severity measures provided distinct information about the patient’s gait. Specifically, the maximal LE_L_ showed no correlation with PSPRS (rho = 0.04, p = 0.81) or PSPRS-GM (rho = 0.1, p = 0.54). Similarly, the risk of falling, as indicated by the LE_S_, did not correlate with PSPRS (rho ≈ 0, p = 0.96) or PSPRS-GM (rho = 0.07, p = 0.68). Additionally, local gait stability, measured by the mean FM, exhibited no correlation with PSPRS (rho ≈ 0.04, p = 0.79) or PSPRS-GM (rho = 0.08, p = 0.62). These findings suggested that LF metrics measured dynamic instability that was not specifically captured in clinical scales. Conversely, LF metrics were not appropriate for assessing overall disease severity in PSP patients. Rather, both metrics were useful and would add greatly to the quantification of the patient’s health status.

## IV. Discussion

The current study modeled the M/L movement of the CoM relative to the CoP as an inverted pendulum. The system’s stable point represented standing still, and “walking” was defined as oscillations around this point [31]. The ground reaction forces applied to this system maintain its stability, at least for healthy human gait. However, PSP affected gait exhibited lateral instability [8], [32] making the analysis of dynamic stability in the M/L direction particularly important for individuals with PSP.

The results of the current study indicated that individuals with PSP experience dynamic instability, and their gait was less tolerant to perturbation compared to able-bodied individuals. While healthy gait stability and tolerance to perturbation decreased with age, PSP-affected gait demonstrated a greater degree of instability independent of age. This finding indicated that the dynamic instability in PSP-affected gait as measured by FM was not secondary to age and was greater than what would be explained by age alone. Additionally, the risk of falls in PSP-affected gait was found to be highly sensitive to cadence. Individuals with PSP and greater cadence (more steps per minute) were more dynamically unstable. This finding aligned with clinical observation in individuals with PSP who occasionally had a rapid uncontrolled small step that often culminated in a fall [13], [33].

The maximal LE for a system described its ability to overcome perturbations, and the average exponential rate of divergence or convergence of its trajectories [15], [34]. A positive LE indicated that, post perturbation, the system will diverge from its nearby trajectories, while a negative LE indicates convergence [35]. Therefore, in a system with LE < 0, a perturbation applied to the system would decay eventually. Although, most participants in the current study exhibited a LE_L_ < 0, the dynamical system approximation for the PSP affected gait yielded a greater LE_L_ compared to healthy gait, indicating a diminished ability to overcome gait perturbations. External perturbations (e.g., trips) and the inability to overcome them during gait caused instability and may lead to falls [36]. The result from the current study provided quantitative proof in support of this observation.

The correlation between LE_S_ and the risk of falling was established for the elderly population [19], [37]. Patients affected by PSP are also known to be at a greater risk of falling [33], [38]. There is currently no literature on the values of LE_S_ for PSP affected gait. The data in the current study showed that the healthy elderly participants and the participants affected by PSP had similar values of LE_S_. Hence LEs alone were not able to distinguish the fall risk among this population. Fall risk was measured by combining LEs with cadence such that LEs increased with increasing cadence among those with PSP. Bruijn et al.’s study showed that, for healthy gait, LE_S_ did not change significantly in the M/L direction with gait speed [39]. The results of the current study agreed with this finding for the control group only. In the PSP group, those who walked at slower speeds had reduced LE_S_ values. A similar relationship between cadence and frequency of falls in patients affected by PSP was discussed by Lindemann et al. [40]. Patients with PSP are well known to have a reduced cadence at their preferred speed of walking [41]. These findings highlighted the importance of reduced cadence as a possible compensatory change to reduce fall risk and maintain dynamic stability, particularly for individuals with PSP.

The participants in the PSP group had a more unstable gait compared to the control group. While gait stability in PSP patients was investigated in prior studies, to our knowledge, this study is the first to employ FM for assessing gait stability in this population. Amano et al. reported that patients with PSP increased their step width in an effort to increase the margin of spatial stability [32]. In another study, the double support time was used to indicate dynamic stability, however this was an indirect measure, assuming that double support time would be greater due to dynamic instability, but this metric could be affected by other issues such as limb pain [8]. The values of FM in the current study indicated that the gait of patients with PSP was more unstable compared to healthy participants.

PSPRS is a disease severity scale that is used by neurologists to grade the patients on various signs and symptoms related to PSP. The gait midline section of PSPRS (PSPRS-GM) grades patients on a broad range of symptoms related to gait (e.g., postural stability using pull test, neck rigidity, etc.) [42]. The LF metrics purely represent gait instability as opposed to clinical scales, such as PSPRS, that are an aggregated score of various motor and non-motor domains. Hence it is not surprising that there was a lack of correlation between these two modalities.

In the current research we demonstrated that LF metrics captured PSP related gait instability and vulnerability to perturbation independent of age. However, the literature presented inconsistent recommendations on using LE to describe gait affected by parkinsonism. Most existing work was performed in PD. Even though maximal LE_L_ had construct validity, this validity was lacking in observational studies [43]. Lahrimi’s study reported that the LE_L_ in PD affected gait was greater than in healthy older adults, though the sample size was small (N = 5 per group) [44]. In contrast, Torres-Pardo et al.’s study, which had a larger sample size comparable to the current study (34 healthy and 42 participants diagnosed with PD), was inconclusive about the use of LE [45]. They observed that PD affected gait exhibited a greater value of LE_L_ and a reduced value of LE_S_ compared to healthy age-matched individuals. Maximal LE_S_ had demonstrated predictive validity in observational studies [43]. However, Torres-Pardo et al.’s statement that “the gait of Parkinson’s disease patients is more stable than that of healthy controls” requires further investigation. These conflicting results highlighted the need for additional research with larger sample sizes to better understand the role of LE in characterizing gait disturbances associated with parkinsonism.

The current study used values of long-term LE calculated analytically. The values of long term and short term LE used in literature were generally calculated using numerical approximation techniques [46]. These values of LE were generally greater than 0, indicating poor perturbation tolerance and high susceptibility to falls, even in healthy adults [16], [26], [46]. An analytical approach to calculate LE_L_ and FM was recently discussed [1]. This analytical approach was shown to result in values of LE_L_, and FM that were closer to the observed gait characteristics.

### A. Study Limitations

A large dataset is essential for obtaining statistically precise estimates of LE [43]. For example, Bruijn et al. reported better precision in their estimates of LE at 150 strides [47]. The current study included walking trials that were relatively short (10 m each). A stitching procedure was performed to create a longer data set (consisting of 19 gait cycles on average) and the LE_L_ was calculated using an analytical method. This approach has been validated previously [1]. Sloot et al. also performed a similar study on healthy adults with impaired balance control due to galvanic vestibular stimulations and concluded that LE_S_ calculated over multiple trials stitched together was a suitable measure to calculate local dynamic stability [28]. In a prior study performed, our group compared various methods for calculating FM and LE in a stitched dataset comprising multiple trials, yielding LE values consistent with those reported in the literature [1]. The goal of the current study was to identify group-level trends and differences in gait behavior between healthy gait and PSP affected gait. Reliable estimates of gait stability using non-linear measures can still be obtained using up to 14 strides, at which point a 30% variability about the median value can be expected [48], [49]. Multiple prior studies have used 10–14 strides to perform reliable analyses of nonlinear stability metrics like Lyapunov exponent analyses [4] [48], [49]. The findings of the current study, when considered alongside the observations listed above, suggested that using shorter trials was not a major limitation.

### B. Future Work

The LE_S_ could be investigated as a metric for quantifying fall risk in patients with PSP. Correlation between the disease severity scales used as outcome measures for PSP (e.g., PSPRS and the Unified Parkinson’s disease rating scale) to the metrics of stability, perturbation tolerance, and risk of falls should also be investigated. Together, these avenues will contribute to a more comprehensive and adaptive framework for assessing and improving gait stability in patients with PSP.

## V. Conclusion

This study highlighted key differences in gait stability between healthy older individuals and patients with PSP using the Lyapunov Floquet theory. PSP affected gait was observed to be more unstable and have reduced tolerance to perturbations compared to healthy gait. The fall risk in patients with PSP was also observed to be sensitive to their cadence. These findings support the need for future research on PSP affected gait and its stability using quantitative non-linear measures.

## Figures and Tables

**TABLE I:** Participant Demographics (Mean ± Std. [range])

	Control group	PSP group	Statistics
**N [Male/Female]**	33 [15/18]	40 [19/21]	
** Age (years)**	64 ± 10 [51 to 82]	71 ± 7 [58 to 86]	p = 0.001
** Height (m)**	1.7 ± 9.2 [1.5 to 1.9]	1.7 ± 9 [1.5 to 1.9]	p = 0.283
**Body Mass (kg)**	77.8 ± 13 [54.6 to 102.6]	76.3 ± 18.5 [48.6 to 117.8]	p = 0.69
**Cadence (steps/min)**	55 ± 4 [48 to 61]	50 ± 5 [38 to 59]	p < 0.001
**PSPRS**	-	35 ± 12 [12 to 58]	-
**PSPRS-GM**	-	10 ± 4 [2 to 16]	-

## References

[1] Bhat SG and Kaufman KR, “Dynamical systems theory applied to short walking trials,” J. Biomechanics, vol. 176, Nov. 2024, Art. no. 112331. 10.1016/j.jbiomech.2024.112331 39340973

[2] Steele JC, Richardson JC, and Olszewski J, “Progressive supranuclear palsy: A heterogeneous degeneration involving the brain stem, basal ganglia and cerebellum with vertical gaze and pseudobulbar palsy, nuchal dystonia and dementia,” Arch. Neurol, vol. 10, no. 4, pp. 333–359, 1964. 14107684 10.1001/archneur.1964.00460160003001

[3] Litvan I. , “Clinical research criteria for the diagnosis of progressive supranuclear palsy (Steele-Richardson-Olszewski syndrome): Report of the NINDS-SPSP international workshop,” Neurology, vol. 47, no. 1, pp. 1–9, Jul. 1996. 8710059 10.1212/wnl.47.1.1

[4] Mahale RR, Krishnan S, Divya KP, Jisha VT, and Kishore A, “Subtypes of PSP and prognosis: A retrospective analysis,” Ann. Indian Acad. Neurol, vol. 24, no. 1, pp. 56–62, 2021. 33911380 10.4103/aian.AIAN_611_20 PMC8061531

[5] Höglinger GU , “Clinical diagnosis of progressive supranuclear palsy: The movement disorder society criteria,” Movement Disorders, vol. 32, no. 6, pp. 853–864, 2017. 28467028 10.1002/mds.26987 PMC5516529

[6] Burn DJ and Lees AJ, “Progressive supranuclear palsy: Where are we now?,” Lancet Neurol, vol. 1, no. 6, pp. 359–369, Oct. 2002. 12849397 10.1016/s1474-4422(02)00161-8

[7] Owolabi L, “Progressive supranuclear palsy misdiagnosed as Parkinson’s disease: A case report and review of literature,” Ann. Med. health Sci. Res, vol. 3, no. 5, p. 44, 2013. 10.4103/2141-9248.121221 PMC3853608 24349849

[8] Amboni M. , “Gait analysis may distinguish progressive supranuclear palsy and Parkinson disease since the earliest stages,” Sci. Rep, vol. 11, no. 1, p. 9297, Apr. 2021. 33927317 10.1038/s41598-021-88877-2 PMC8084977

[9] Ricciardi C. , “Using gait analysis’ parameters to classify Parkinsonism: A data mining approach,” Comput. Methods Programs Biomed, vol. 180, Oct. 2019, Art. no. 105033. 10.1016/j.cmpb.2019.105033 31445485

[10] Ali F, Loushin SR, Botha H, Josephs KA, Whitwell JL, and Kaufman K, “Laboratory based assessment of gait and balance impairment in patients with progressive supranuclear palsy,” J. Neurological Sci, vol. 429, Oct. 2021, Art. no. 118054. 10.1016/j.jns.2021.118054 PMC8489851 34461552

[11] Dale ML, Prewitt AL, Harker GR, McBarron GE, and Mancini M, “Perspective: Balance assessments in progressive supranuclear palsy: Lessons learned,” Frontiers Neurol., vol. 13, Jan. 2022, Art. no. 801291. 10.3389/fneur.2022.801291 PMC8828584 35153996

[12] Zwergal A. , “Postural imbalance and falls in PSP correlate with functional pathology of the thalamus,” Neurology, vol. 77, no. 2, pp. 101–109, Jul. 2011. 21613601 10.1212/WNL.0b013e318223c79d

[13] Brown F, Rowe JB, Passamonti L, and Rittman T, “Falls in progressive supranuclear palsy,” Movement Disorders Clin. Pract, vol. 7, no. 1, pp. 16–24, 2019. 10.1002/mdc3.12879 PMC6962663 31970205

[14] Bhat SG, Subramanian SC, Sugar TS, and Redkar S, “Application of floquet theory to human gait kinematics and dynamics,” J. Mech. Robot, vol. 13, no. 6, Dec. 2021.

[15] Dingwell JB, Cusumano JP, Cavanagh PR, and Sternad D, “Local dynamic stability versus kinematic variability of continuous overground and treadmill walking,” J. Biomechanical Eng, vol. 123, no. 1, pp. 27–32, Feb. 2001. 10.1115/1.1336798 11277298

[16] Caronni A. , “Local dynamic stability of gait in people with early multiple sclerosis and no-to-mild neurological impairment,” IEEE Trans. Neural Syst. Rehabil. Eng, vol. 28, no. 6, pp. 1389–1396, Jun. 2020. 32356754 10.1109/TNSRE.2020.2991636

[17] Chini G. , “Local stability of the trunk in patients with degenerative cerebellar ataxia during walking,” Cerebellum, vol. 16, no. 1, pp. 26–33, Feb. 2017. 26811155 10.1007/s12311-016-0760-6

[18] Reynard F, Vuadens P, Deriaz O, and Terrier P, “Could local dynamic stability serve as an early predictor of falls in patients with moderate neurological gait disorders? A reliability and comparison study in healthy individuals and in patients with paresis of the lower extremities,” PLoS ONE, vol. 9, no. 6, Jun. 2014, Art. no. e100550. 10.1371/journal.pone.0100550 PMC4065053 24949737

[19] Lockhart TE and Liu J, “Differentiating fall-prone and healthy adults using local dynamic stability,” Ergonomics, vol. 51, no. 12, pp. 1860–1872, Dec. 2008. 19034782 10.1080/00140130802567079 PMC2892176

[20] Teschl G, Ordinary Differential Equations and Dynamical Systems. Providence, RI, USA: American Mathematical Soc., 2012, p. 93.

[21] Bruijn SM, Bregman DJJ, Meijer OG, Beek PJ, and van Dieën JH, “Maximum Lyapunov exponents as predictors of global gait stability: A modelling approach,” Med. Eng. Phys, vol. 34, no. 4, pp. 428–436, May 2012. 21856204 10.1016/j.medengphy.2011.07.024

[22] Su JL-S and Dingwell JB, “Dynamic stability of passive dynamic walking on an irregular surface,” J. Biomech. Eng, vol. 129, no. 6, pp.802–810, Dec. 2007, doi:10.1115/1.2800760. 18067383

[23] Kurz MJ, Markopoulou K, and Stergiou N, “Attractor divergence as a metric for assessing walking balance,” Nonlinear Dynamics Psychol. Life Sci, vol. 14, no. 2, pp.151–164, Apr. 2010. 20346260

[24] Chang MD, Sejdić E, Wright V, and Chau T, “Measures of dynamic stability: Detecting differences between walking overground and on a compliant surface,” Human Movement Sci, vol. 29, no. 6, pp. 977–986, Dec. 2010. 10.1016/j.humov.2010.04.009 20655606

[25] Rezaei A, Bhat SG, Cheng C-H, Pignolo RJ, Lu L, and Kaufman KR, “Age-related changes in gait, balance, and strength parameters: A cross-sectional study,” PLoS ONE, vol. 19, no. 10, Oct. 2024, Art. no. e0310764. 10.1371/journal.pone.0310764 PMC11498712 39441815

[26] Granata KP and Lockhart TE, “Dynamic stability differences in fall-prone and healthy adults,” J. Electromyogr. Kinesiol, vol. 18, no. 2, pp. 172–178, Apr. 2008. 17686633 10.1016/j.jelekin.2007.06.008 PMC2895268

[27] Winter DA, Biomechanics and Motor Control of Human Movement. Hoboken, NJ, USA: Wiley, 2009.

[28] Sloot LH, van Schooten KS, Bruijn SM, Kingma H, Pijnappels M, and van Dieën JH, “Sensitivity of local dynamic stability of over-ground walking to balance impairment due to galvanic vestibular stimulation,” Ann. Biomed. Eng, vol. 39, no. 5, pp. 1563–1569, May 2011. 21222163 10.1007/s10439-010-0240-y PMC3071943

[29] Rosenstein MT, Collins JJ, and De Luca CJ, “A practical method for calculating largest Lyapunov exponents from small data sets,” Phys. D, Nonlinear Phenomena, vol. 65, nos. 1–2, pp. 117–134, May 1993.

[30] (2021). R: A Language and Environment for Statistical Computing. R Foundation for Statistical Computing. [Online]. Available: https://www.R-project.org/

[31] Henders MG and Soudack AC, “Dynamics and stability state-space of a controlled inverted pendulum,” Int. J. Non-Linear Mech, vol. 31, no. 2, pp. 215–227, Mar. 1996.

[32] Amano S. , “Discriminating features of gait performance in progressive supranuclear palsy,” Parkinsonism Rel. Disorders, vol. 21, no. 8, pp. 888–893, Aug. 2015. 10.1016/j.parkreldis.2015.05.017 26032992

[33] Bluett B. , “Understanding falls in progressive supranuclear palsy,” Parkinsonism Rel. Disorders, vol. 35, pp. 75–81, Feb. 2016, doi: 10.1016/j.parkreldis.2016.12.009. 28007518

[34] Wolf A, “13 Quantifying chaos with Lyapunov exponents,” in Chaos A. V. Holden, Ed.,Princeton, NJ, USA: Princeton Univ. Press, 1986, pp. 273–290. [Online]. Available: 10.1515/9781400858156.273

[35] Sandri M, “Numerical calculation of Lyapunov exponents,” Mathematica J, vol. 6, no. 3, pp. 78–84, 1996.

[36] Gerards MHG, Marcellis RGJ, Poeze M, Lenssen AF, Meijer K, and de Bie RA, “Perturbation-based balance training to improve balance control and reduce falls in older adults – study protocol for a randomized controlled trial,” BMC Geriatrics, vol. 21, no. 1, pp. 1–12, Dec. 2021. 33407204 10.1186/s12877-020-01944-7 PMC7788687

[37] Toebes MJP, Hoozemans MJM, Furrer R, Dekker J, and van Dieën JH, “Local dynamic stability and variability of gait are associated with fall history in elderly subjects,” Gait Posture, vol. 36, no. 3, pp. 527–531, Jul. 2012. 22748312 10.1016/j.gaitpost.2012.05.016

[38] Kim S-L, Lee M-J, and Lee M-S, “Cognitive dysfunction associated with falls in progressive supranuclear palsy,” Gait Posture, vol. 40, no. 4, pp. 605–609, Sep. 2014. 25088758 10.1016/j.gaitpost.2014.07.005

[39] Bruijn SM, van Dieën JH, Meijer OG, and Beek PJ, “Is slow walking more stable?,” J. Biomechanics, vol. 42, no. 10, pp. 1506–1512, Jul. 2009. 10.1016/j.jbiomech.2009.03.047 19446294

[40] Lindemann U. , “Clinical and dual-tasking aspects in frequent and infrequent fallers with progressive supranuclear palsy,” Movement Disorders, vol. 25, no. 8, pp. 1040–1046, Jun. 2010. 20131396 10.1002/mds.23023

[41] Egerton T, Williams DR, and Iansek R, “Comparison of gait in progressive supranuclear palsy, Parkinson’s disease and healthy older adults,” BMC Neurol, vol. 12, no. 1, pp. 1–6, Dec. 2012. 23031506 10.1186/1471-2377-12-116 PMC3517411

[42] Golbe LI and Ohman-Strickland PA, “A clinical rating scale for progressive supranuclear palsy,” Brain, vol. 130, no. 6, pp. 1552–1565, Apr. 2007. 17405767 10.1093/brain/awm032

[43] Bruijn SM, Meijer OG, Beek PJ, and van Dieën JH, “Assessing the stability of human locomotion: A review of current measures,” J. Roy. Soc. Interface, vol. 10, no. 83, Jun. 2013, Art. no. 20120999. 10.1098/rsif.2012.0999 PMC3645408 23516062

[44] Lahmiri S, “Gait nonlinear patterns related to Parkinson’s disease and age,” IEEE Trans. Instrum. Meas, vol. 68, no. 7, pp. 2545–2551, Jul. 2019.

[45] Torres-Pardo A. , “Is Lyapunov exponent a reliable metric to detect dynamic stability in Parkinson’s disease?,” in Proc. 45th Annu. Int. Conf. IEEE Eng. Med. Biol. Soc. (EMBC), Jul. 2023, pp. 1–4. [Online]. Available: 10.1109/EMBC40787.2023.10341028 38083092

[46] Mehdizadeh S, “The largest Lyapunov exponent of gait in young and elderly individuals: A systematic review,” Gait Posture, vol. 60, pp. 241–250, Feb. 2018. 29304432 10.1016/j.gaitpost.2017.12.016

[47] Bruijn SM, van Dieën JH, Meijer OG, and Beek PJ, “Statistical precision and sensitivity of measures of dynamic gait stability,” J. Neurosci. Methods, vol. 178, no. 2, pp. 327–333, Apr. 2009. 19135478 10.1016/j.jneumeth.2008.12.015

[48] Bisi M, Riva F, and Stagni R, “Measures of gait stability: Performance on adults and toddlers at the beginning of independent walking,” J. NeuroEng. Rehabil, vol. 11, no. 1, p. 131, 2014. 25186796 10.1186/1743-0003-11-131 PMC4163161

[49] Riva F, Bisi MC, and Stagni R, “Gait variability and stability measures: Minimum number of strides and within-session reliability,” Comput. Biol. Med, vol. 50, pp. 9–13, Jul. 2014. 24792493 10.1016/j.compbiomed.2014.04.001

